# Worldwide prevalence rates and trends of eczema in youth

**Published:** 2023-03

**FEBRUARY 8, 2023** - The results come from an analysis of data from 14 countries involving 74,361 adolescents aged 13–14 years and 47,907 children aged 6–7 years.

Investigators estimated an average increase over 27 years in the prevalence of current eczema symptoms of 0.98% per decade in adolescents and 1.21% per decade in children, and of 0.26% and 0.23% per decade in severe eczema symptoms. However, there was substantial variation in changes in eczema prevalence over time by income and region.

“Eczema remains a big public health problem around the world,” said corresponding author Sinéad Langan, PhD, of the London School of Hygiene & Tropical Medicine. “Global research efforts are needed to address the burden related to eczema with continued international efforts to identify strategies to prevent the onset of eczema and to better manage the impact on individuals, their families, and health service.”

***Link to Study: https://onlinelibrary.wiley.com/doi/10.1111/cea.14276***

***Full citation:** “Trends in eczema prevalence in children and adolescents: A Global Asthma Network Phase I Study.” Sinéad Máire Langan, Amy R. Mulick, Charlotte E. Rutter, Richard J. Silverwood, Innes Asher, Luis García-Marcos, Eamon Ellwood, Karen Bissell, Chen-Yuan Chiang, Asma El Sony, Philippa Ellwood, Guy B. Marks, Kevin Mortimer, A. Elena Martínez-Torres, Eva Morales, Virginia Perez-Fernandez, Steven Robertson, Hywel C. Williams, David P. Strachan, Neil Pearce, the Global Asthma Network Phase I Study Group. Clin Exp Allergy; Published Online: 08 February 2023 (DOI: 10.1111/cea.14276).*

*Copyright © 2019 The Cochrane Collaboration. Published by John Wiley & Sons, Ltd., reproduced with permission.*

